# Editorial: Education in obstetrics and gynecology: 2025

**DOI:** 10.3389/fmed.2026.1934033

**Published:** 2026-07-29

**Authors:** Florian Recker, Shavi Fernando, Glenn D. Posner

**Affiliations:** 1 Department of Obstetrics, Faculty of Medicine and University Hospital Cologne, University of Cologne, Cologne, Germany; 2 Department of Obstetrics and Gynecology, Faculty of Medicine, Nursing and Health Sciences, Monash University, Melbourne, VIC, Australia; 3 Department of Obstetrics and Gynecology, Faculty of Medicine, University of Ottawa, Ottawa, ON, Canada

**Keywords:** assessment, competency-based medical education, health professions education, medical education, obstetrics and gynecology, simulation-based training, virtual reality, workforce

## Introduction

The obstetric and gynecological (OB/GYN) workforce carries a disproportionate share of the global health burden. The specialty sits at the center of two of the most closely watched indicators of a functioning health system—maternal mortality and reproductive health outcomes—yet it operates under increasing challenges including, but not limited to, expanding clinical knowledge, contracting training time, workforce shortages, and rising expectations that clinicians be simultaneously technically excellent and humanistically attuned. How we educate the next generation of obstetricians, gynecologists, midwives, and nurses is therefore not a parochial concern for medical educators; it is a determinant of population health.

This Research Topic is the second volume in a series that began with our inaugural collection (1). In the interval between the two volumes, the field has moved quickly. Contemporary syntheses now describe OB/GYN education as being in a genuinely transformative phase—reshaped by simulation, competency-based frameworks, digital and tele-education, and an increasingly explicit equity agenda—while cautioning that access to these innovations remains deeply unequal, particularly across low- and middle-income settings (2). The 11 original research articles gathered here map that landscape with unusual breadth. They span the full educational continuum, from preclinical undergraduates to residents and subspecialty fellows; they include medical, midwifery, and nursing students; and they report data from Germany, China, Pakistan, Saudi Arabia, Canada, and a multi-country European consortium. Read together, they offer both a snapshot of where the evidence now stands and a collection of signposts for where it needs to go.

## Simulation and immersive technologies: from technical drills to the whole clinician

The single largest theme in this Research Topic is simulation, and this reflects the maturity of the underlying evidence. The foundational meta-analysis by Cook et al. established more than a decade ago that technology-enhanced simulation is reliably associated with improvements in knowledge, skills, and behaviors relative to no intervention (3), and a recent OB/GYN-specific meta-analysis of 30 randomized controlled trials has confirmed substantial gains in surgical skill, operative time, and learner confidence—notably finding that high-fidelity virtual reality simulators and low-fidelity box trainers perform comparably (4). The contributions here push this established base into new populations and, importantly, into new outcome domains.

Three studies address simulation in the pre-registration medical curriculum. Mohsin et al. used a mixed-methods design to show that simulation-based teaching in gynecology and obstetrics improved both measured learning and student engagement, with qualitative data illuminating why learners valued the approach. Adams, Stosch et al., in a study aptly titled “Bridging the gap,” demonstrated that simulation-based OB/GYN training improved not only objectively assessed skills but also final-year students' self-perceived preparedness—a reminder that confidence and competence are related but distinct educational targets. Most intriguingly, Yao et al. moved simulation beyond the technical: using an unfolding case-based design during OB/GYN internships, they reported gains in students' caring ability and caring behaviors. That simulation can be harnessed to cultivate the relational and affective dimensions of care, and not only procedural dexterity, is an important corrective to a field that has sometimes equated fidelity with psychomotor realism alone.

A parallel set of studies interrogates immersive and manufactured technologies. Adams, Klein et al. evaluated V.T.O.B.S., a virtual-reality environment for learning birth mechanics, in a controlled cohort of undergraduates—a use case in which VR's capacity to render dynamic three-dimensional spatial relationships is especially well matched to the learning problem. Sa-Couto et al., working across the multicenter PROGRESSION consortium, tested a VR-enhanced program for maternal positioning in midwifery students and documented measurable pre–post gains, extending immersive learning into interprofessional and midwifery training where the evidence base has been thinner. Dumont et al. took a different technological route, integrating 3D-printed simulation models into a pediatric and adolescent gynecology (PAG) fellowship and reporting improved trainee confidence alongside strong curriculum acceptability—a demonstration that low-cost, reproducible physical models can fill training gaps in subspecialties where patient volumes are low and hands-on exposure is scarce. These findings sit comfortably within the emerging systematic-review literature on VR in obstetric procedures (5), but they also inherit its principal caveat: immersive and manufactured solutions remain dependent on infrastructure, technical support, and faculty capacity, and their promise for democratizing training is real only where those enablers exist.

## Assessment, cognitive integration, and competency

If simulation dominates the “how” of teaching, two contributions engage the equally consequential question of assessment and cognitive design. Chen et al. combined a student standardized-patient model with structured peer assessment and showed improved SOAP-note proficiency among OB/GYN residents—an elegant example of using learners themselves as both instructional resource and assessors, with implications for programs operating under supervisory constraints. Ismail-Khan et al. contributed one of the methodologically strongest studies in the Research Topic: a multi-center cluster-randomized controlled trial demonstrating that the instructional modality used to vertically integrate anatomy shapes both cognitive load and performance on an operative-interpretation task. Cluster-randomized designs remain rare in health-professions education, and this study is a welcome signal that OB/GYN education research is willing to meet a higher evidentiary bar. Both papers speak to the wider migration toward competency-based medical education, whose implementation across specialties remains uneven and incompletely evaluated (6); rigorous, mechanism-focused studies of this kind are exactly what that transition requires.

## Digital learning after the pandemic

The pandemic-era pivot to digital education has left a durable question: not whether to use digital modalities, but how. Cirkel et al. surveyed medical students' preferences regarding digital teaching in OB/GYN and found—consistent with a maturing literature—that learners value digital formats as a complement to, rather than a replacement for, hands-on and in-person learning. The practical implication is a blended model in which digital tools are deployed for what they do best—flexibility, reach, and foundational knowledge—while the experiential core of clinical training is protected.

## The workforce pipeline: career choice, gender, and the learning environment

Finally, two studies turn from pedagogy to the human ecology of the specialty. Lone et al. examined the factors influencing and discouraging medical students in Eastern Saudi Arabia from choosing OB/GYN as a career—a question with direct bearing on a specialty facing workforce pressures in many health systems. Bai et al. investigated patient acceptance of medical students in OB/GYN practice in China and reported that gender, but not nationality, shaped patients' willingness to permit student involvement. This is a subtle but important finding: patient acceptance is a hidden gatekeeper of clinical exposure, and if it varies systematically by learner characteristics, it can quietly distort who gets to learn. Both studies remind us that curricula do not operate in a vacuum; the clinical learning environment, career perceptions, and patient attitudes together determine who enters the specialty and how well they are trained once there.

## Synthesis and future directions

Several threads run across these 11 papers. The first is geographic breadth: the conversation about OB/GYN education is now genuinely global, and the flow of methodological innovation is no longer unidirectional. The second is methodological maturation—this Research Topic includes mixed-methods studies, controlled cohorts, quasi-experiments, and a cluster-randomized trial, a heartening trajectory for a field long dominated by single-institution descriptive surveys. The third, and perhaps most important, is a recurring conceptual message: technology augments but does not replace. Whether the outcome is caring behavior (Yao et al.), self-perceived preparedness (Adams, Stosch et al.), or patient acceptance (Bai et al.), the human dimension of medical education keeps reasserting itself even in studies built around the most advanced tools.

Three priorities follow for the next volume of this work. First, the field must climb the outcomes hierarchy: most studies here, in keeping with the broader literature, report learner-level outcomes such as skills, confidence, and satisfaction, whereas the questions that matter most—retention of competence over time, transfer to independent clinical performance, and ultimately patient outcomes—remain comparatively underexplored (7). Second, progress will depend on shared, validated assessment instruments and on the kind of multi-institutional and international collaboration that the PROGRESSION consortium (Sa-Couto et al.) and the multi-center trial reported here (Ismail-Khan et al.) exemplify; without common metrics, individual studies cannot be pooled and the field cannot cumulate. Third, the convergence of artificial intelligence with immersive technologies—adaptive learning pathways, automated and personalized feedback, and AI-augmented simulation—is the most plausible near-term frontier, and it will demand the same evidentiary discipline these authors have modeled rather than uncritical enthusiasm. Alongside these technical directions, the workforce and equity questions raised here deserve sustained attention: the finest curriculum is of little value if too few trainees choose the specialty, or if access to innovation is confined to well-resourced centers.

We thank the authors for their contributions, the reviewers whose careful work strengthened every paper, and the readers who make a Research Topic like this worthwhile. The education of those who care for women and birthing people is unfinished work, and we hope this volume is a useful step in it.
